# Congenital Tuberculosis

**Published:** 1903-01-24

**Authors:** 


### CONGENITAL TUBERCULOSIS.

At a recent meeting of the Pathological Society, Dr. Andrewes exhibited the lungs of an infant affected with congenital tuberculosis, a condition which, although very rare in the human subject, is well known to occur in certain of the lower animals. The infant from whom the specimen had been taken was stated to have died shortly after birth, and this was confirmed by the small size of the organs and by the fact that the ductus arteriosus was still pervious. The tracheal and bronchial glands were enlarged and caseous, a condition of affairs which must evidently have arisen long before birth, while the lungs contained numerous miliary tubercules.

The histological appearances were quite characteristic of tubercle. As to the question how the infection had arisen, it was suggested that it had been caused by the fetus swallowing liquor amnii containing tubercle bacilli. These, it was said, might have been derived from a disintegrating lesion of the placenta, for it is known that the entrance of amniotic fluid into the mouth may take place during fetal life.

